# *Hox* genes reveal variations in the genomic DNA of allotetraploid hybrids derived from *Carassius auratus* red var. (female) × *Cyprinus carpio* L*.* (male)

**DOI:** 10.1186/s12863-020-0823-z

**Published:** 2020-03-04

**Authors:** Rurong Zhao, Yude Wang, Li Zou, Yaxin Luo, Huifang Tan, Jiajun Yao, Minghe Zhang, Shaojun Liu

**Affiliations:** 1 State Key Laboratory of Developmental Biology of Freshwater Fish, Hunan Normal University, Changsha, 410081 Hunan People’s Republic of China; 2 College of Life Sciences, Hunan Normal University, Changsha, 410081 Hunan People’s Republic of China; 3 Fisheries Research Institute of Hunan Province, Changsha, 410153 People’s Republic of China

**Keywords:** PCR survey, *Hox* gene, Allotetraploid hybrid, Evolution

## Abstract

**Background:**

*Hox* transcription factors are master regulators of animal development. Although highly conserved, they can contribute to the formation of novel biological characteristics when modified, such as during the generation of hybrid species, thus potentially serving as species-specific molecular markers*.* Here, we systematically studied the evolution of genomic sequences of *Hox* loci in an artificial allotetraploid lineage (4nAT, 4n = 200) derived from a red crucian carp (♀, RCC, 2n = 100) × common carp (♂, CC, 2n = 100) cross and its parents (RCC and CC).

**Results:**

PCR amplification yielded 23 distinct *Hox* gene fragments from 160 clones in 4nAT, 22 fragments from 90 clones in RCC, and 19 fragments from 90 clones in CC. Sequence alignment of the *HoxA3a* and *HoxC10a* genes indicated both the inheritance and loss of paternal genomic DNA in 4nAT. The *HoxA5a* gene from 4nAT consisted of two subtypes from RCC and two subtypes from CC, indicating that homologous recombination occurred in the 4nAT hybrid genome. Moreover, 4nAT carried genomic pseudogenization in the *HoxA10b* and *HoxC13a* loci. Interestingly, a new type of *HoxC9a* gene was found in 4nAT as a hybrid sequence of CC and RCC by recombination in the intronic region.

**Conclusion:**

The results revealed the influence of *Hox* genes during polyploidization in hybrid fish. The data provided insight into the evolution of vertebrate genomes and might be benefit for artificial breeding programs.

## Background

Genome duplication, and therefore polyploidy, can occur during animal evolution [1, 2]. Two types of polyploidy have been identified based on genetic origin: autopolyploidy, which originates from the genome duplication of a single species, and allopolyploidy, which occurs via interspecific hybridization and results in haploid chromosomes inherited from different species. Allopolyploidy is prevalent in nature, suggesting the evolutionary advantage of maintaining genetic heterogeneity [3]. Allopolyploid plays an important role in facilitating the activation of cryptic mobile elements and rapid genomic change [4]. This ‘genomic shock’ has been reported in many allopolyploid plants and occurs through gene loss, chromosome mispairing, retrotransposon activation, altered methylation, or rearrangements between parental genomes. These can lead to novel gene sequences or differential homologous gene expression in hybrids [5]. After a short period of laboratory experiments, chromosmal rearragements, duplications and deletions of chromosome segments, and shifts in ploidy have been observed and these appear to be adaptive [6]. Changes in genome structure usually have a direct impact on the phenotype and can contribute to individual adaptation [7]. Rapid genomic DNA changes have been described in several allopolyploid plants [8]. Compared to plants, animals rarely undergo allopolyploidy. Therefore, the genome variations during the animal allopolyploidy process are unclear [9].

**Fig. 1:**
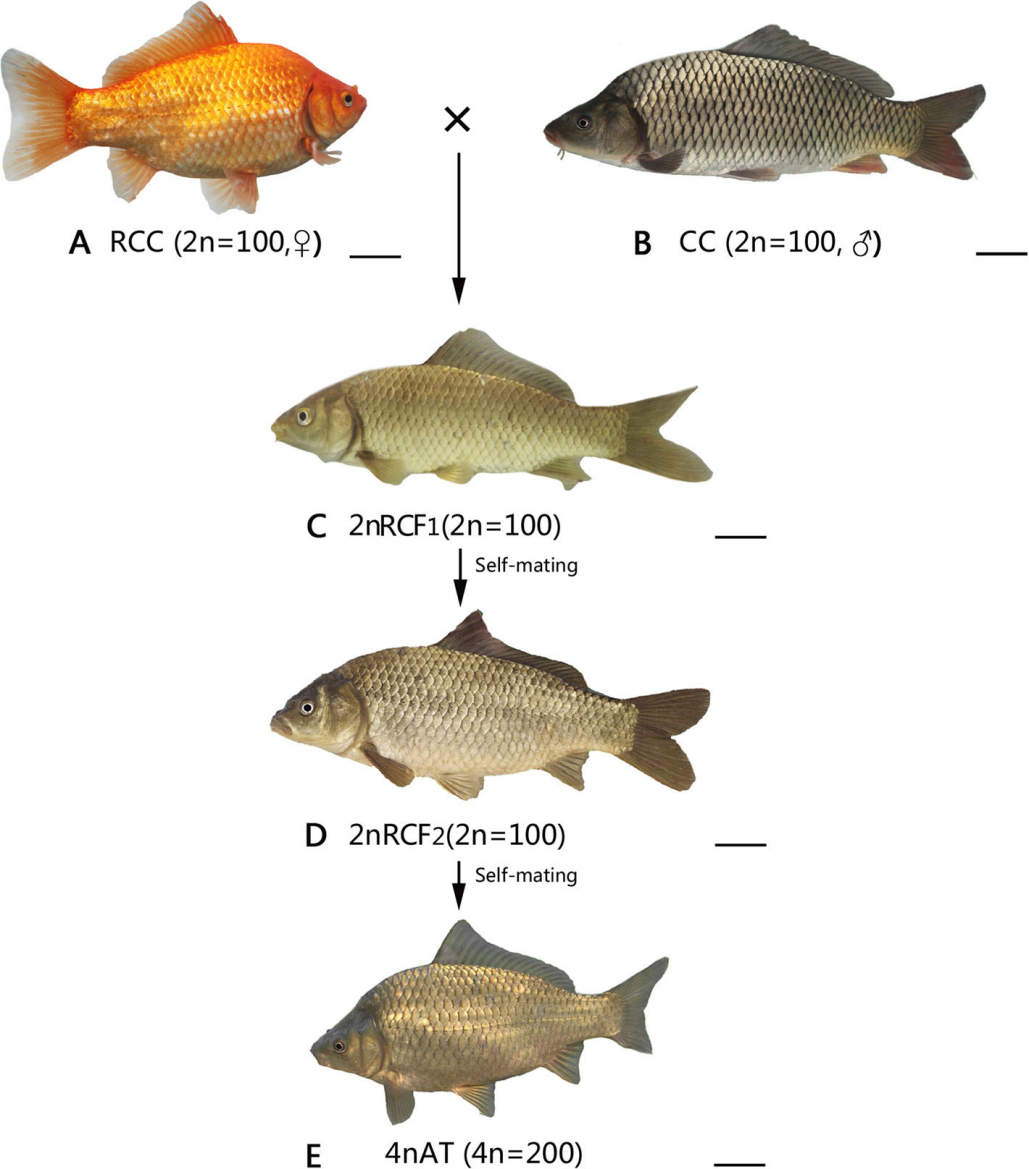
Crossing procedure and appearances of RCC, CC, 2nRCF_1_, 2nRCF_2_ and 4nAT. **a**: RCC; **b**: CC; **c**: 2nRCF_1_; **d**: 2nRCF_2_; **e**: 4nAT. Bar = 3 cm

In a previous study, we obtained fertile allotetraploid hybrids from the interspecific hybridization of red crucian carp (RCC; *Carassius auratus* red var., ♀, 2n = 100) with common carp (CC; *Cyprinus carpio* L., ♂, 2n = 100) [10]. Although the F_1_ and F_2_ progenies were diploid hybrids (2n = 100), fertile allotetraploid offspring of both sexes were produced from the F_3_ and later generations (Fig. 1) [11]. These allotetraploid offspring were used to generate rapidly growing and strongly resistant infertile triploids by hybridization with diploid fishes [12]. Hybrid polyploid fish have wide applications in the Chinese fish industry and they also provide an opportunity to study the molecular and genetic mechanisms that underlie the origination of evolutionary novelties including genome evolution and adaptation. Previous studies confirmed the rapid genomic DNA changes in 4nAT [13], and in this study we investigated the genetic elements susceptible to rapid genomic changes.

*Hox* genes belong to a large family of transcription factors. They are organized in clusters within the genome and are characterized by a homeobox, which encodes a DNA-binding motif known as the homeodomain [14, 15]. *Hox* genes and *Hox* clusters provide evidence that rounds of genome duplications (the one-to-four (−to-eight-to-more in fish) rule) occur in vertebrates [16]. Recent studies have demonstrated that *Hox* gene clusters are fragmented, reduced, or expanded in many animals. These findings correlate with morphological changes occurring during evolution [17]. The *HoxC9* is a regulator of body patterning and development. Its expression has been described in the hind limb blood vessels of mice [18] and the axial vasculature of zebrafish [19]. *HoxC9* may also be involved in the differentiation of bone marrow derived stem cells to endothelial cells because it was upregulated during stem cell differentiation [20]. *Hox* genes are clustered in the common ancestors of chordates, arthropods and nematodes and play an important role in embryogenesis by encoding transcription factors [21]. Therefore, *Hox* genes and *Hox* gene clusters may provide information about the process of evolution at the molecular level [22].

In this study, we compared the *Hox* genes of artificially derived 4nAT lineages with those of the original RCC and CC parents to obtain information on the genomic evolution of allopolyploid animals. Exploration of 4nAT and its related molecular genetic relationship with the parental lines can provide insight into the evolution of selected species. Specific genome replication provides an example for the evolution of fish.

## Results

### Sequence information for RCC, CC, and 4nAT

**Fig. 2:**
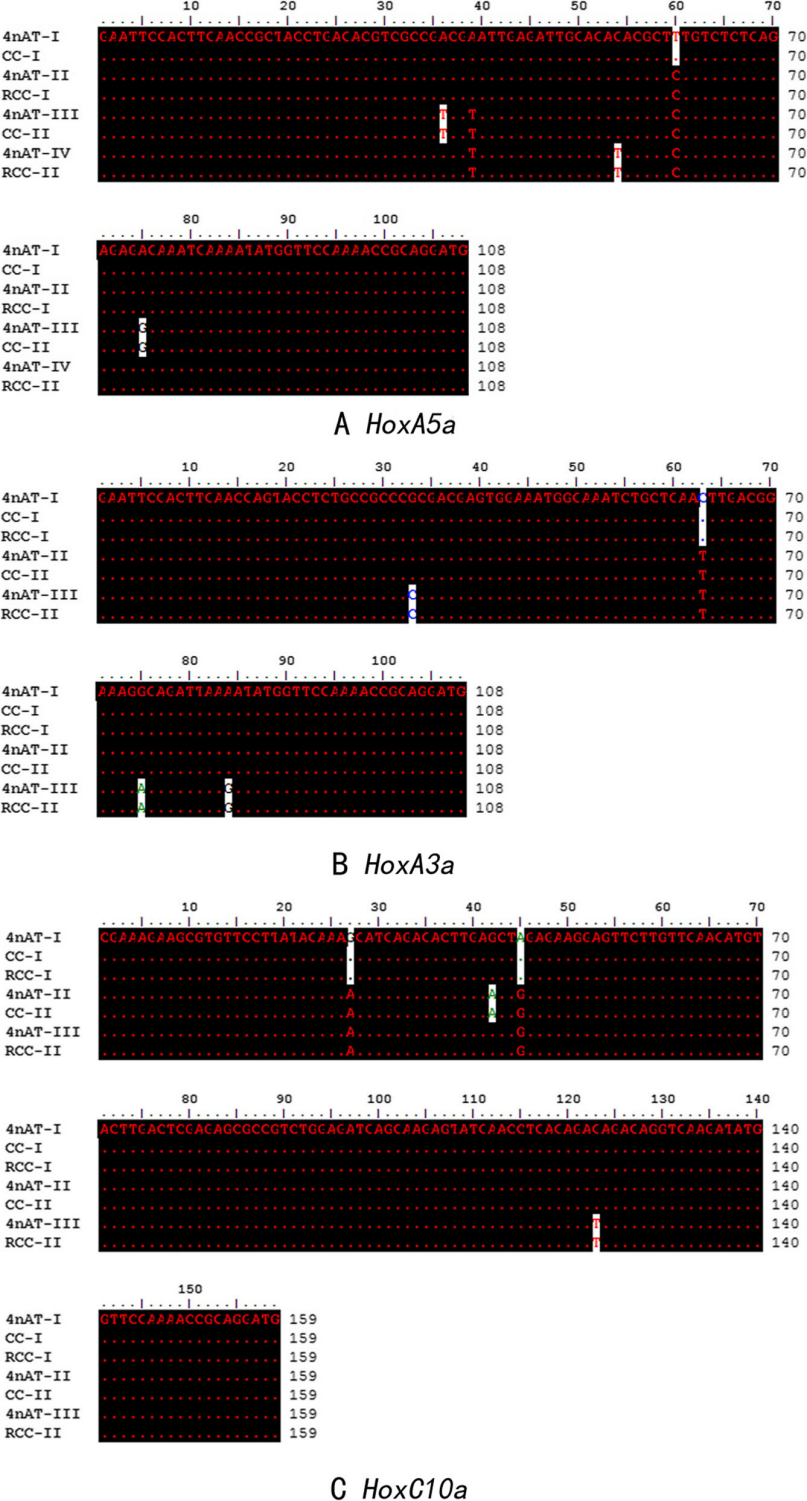
Comparison of the *HoxA5a*, *HoxA3a*, *HoxC10a* sequences from 4nAT, RCC and CC. CC-I and CC-II were two isoforms of *HoxA5a* from CC, RCC-I and RCC-II were two isoforms of *HoxA5a* from RCC, and 4nAT-I, 4nAT-II, 4nAT-III and 4nAT-IV were four isoforms of *HoxA5a* found in 4nAT. Dots indicated identical nucleotides at a given site (**a**). Comparisons of the *HoxA3a* (**b**) and *HoxC10a* (**c**) sequences from 4nAT, RCC and CC were found in B and C, respectively. CC-I and CC-II were two isoforms from CC, RCC-I and RCC-II were from RCC, and 4nAT-I, 4nAT-II and 4nAT-III were found in 4nAT. Dots indicated identical nucleotides at a given site

Using two pairs of degenerate primers, we obtained 90 sequences of PCR clones from RCC, 90 sequences from CC, and 160 sequences from 4nAT. To assign names to the *Hox* genes, all acquired sequences were screened for *Hox* gene fragments using BLAST searches against NCBI and were aligned using Clustal X (2.0) software for verification. According to BLAST searches and sequence alignment analysis, all but 17 sequences could be assigned unambiguously to 29 distinct *Hox* genes. A summary of the analyses and proposed gene identification was presented in Table 1. The combined data of the PCR products yielded 22 distinct *Hox* gene fragments in RCC, 19 in CC, and 23 in 4nAT. Fifteen *Hox* gene fragments were found both in 4nAT and its original parents. Due to amplification bias during PCR amplification, we acquired more clones of *HoxA3a*, *HoxA5a*, and *HoxC10a* gene fragments than from other *Hox* genes. These *Hox* sequences were analyzed further. There were four isoforms in 4nAT (4nAT-I, 4nAT-II, 4nAT-III, and 4nAT-IV), two isoforms in RCC (RCC-I and RCC-II) and two isoforms in CC (CC-I and CC-II). Among the four isoforms of the *HoxA5a* gene, two were consistent with the *HoxA5a* gene in CC and the other two were consistent with the *HoxA5a* genes in RCC (Fig. 2a). The similarities between 4nAT-I and CC-I, 4nAT-II and RCC-I, 4nAT-III and CC-II, and 4nAT-IV and RCC-II were all 100%. We detected two isoforms of *HoxA3a* and *HoxC10a* in the genome of CC and RCC. However, we observed only three isoforms of *HoxA3a* (Fig. 2b) and *HoxC10a* (Fig. 2c) in 4nAT. For *HoxA3a*, the identities between 4nAT-I and CC-I, 4nAT-I and RCC-I, 4nAT-II and CC-II, and 4nAT-II and RCC-II were all 100%. For *HoxC10a*, the identities between 4nAT-I and CC-I, 4nAT-I and RCC-I, 4nAT-II and CC-II and 4nAT-III and RCC-II were all 100%. Almost all orthologous sequences in both RCC and CC showed a higher percentage of similarity to each other than to their duplicated homeologous genes within species.

**Table 1:** Summary of the PCR fragments from the 4nAT and its original parents

PG	Gene name	The total number of *Hox* gene fragments		
		RCC	CC	4nAT
1	*HoxB1b*	2	1	2
2	*HoxA2b*		1	
3	*HoxA3a*	13	10	16
	*HoxB3a*	7	9	8
4	*HoxA4a*	1		
5	*HoxA5a*	11	15	35
	*HoxC5a*	3	1	4
6	*HoxC6a*	1		
7	*HoxB7a*	4	2	5
8	*HoxB8a*		3	3
9	*HoxA9a*	1		2
	*HoxA9b*	3		
	*HoxB9a*	3	2	5
	*HoxC9a*	5	5	13
10	*HoxA10b*	7	2	5
	*HoxB10a*	1		3
	*HoxC10a*	13	14	22
	*HoxD10a*			1
11	*HoxA11a*	3	2	3
	*HoxA11b*			1
	*HoxC11a*			1
	*HoxD11a*			1
12	*HoxC12a*	2		
	*HoxD12a*		1	1
13	*HoxA13a*	1	3	1
	*HoxA13b*	1	1	3
	*HoxB13a*	1	1	
	*HoxC13a*	4	5	12
	*HoxD13a*	1	5	5
Total		88	83	152

**Fig. 3:**
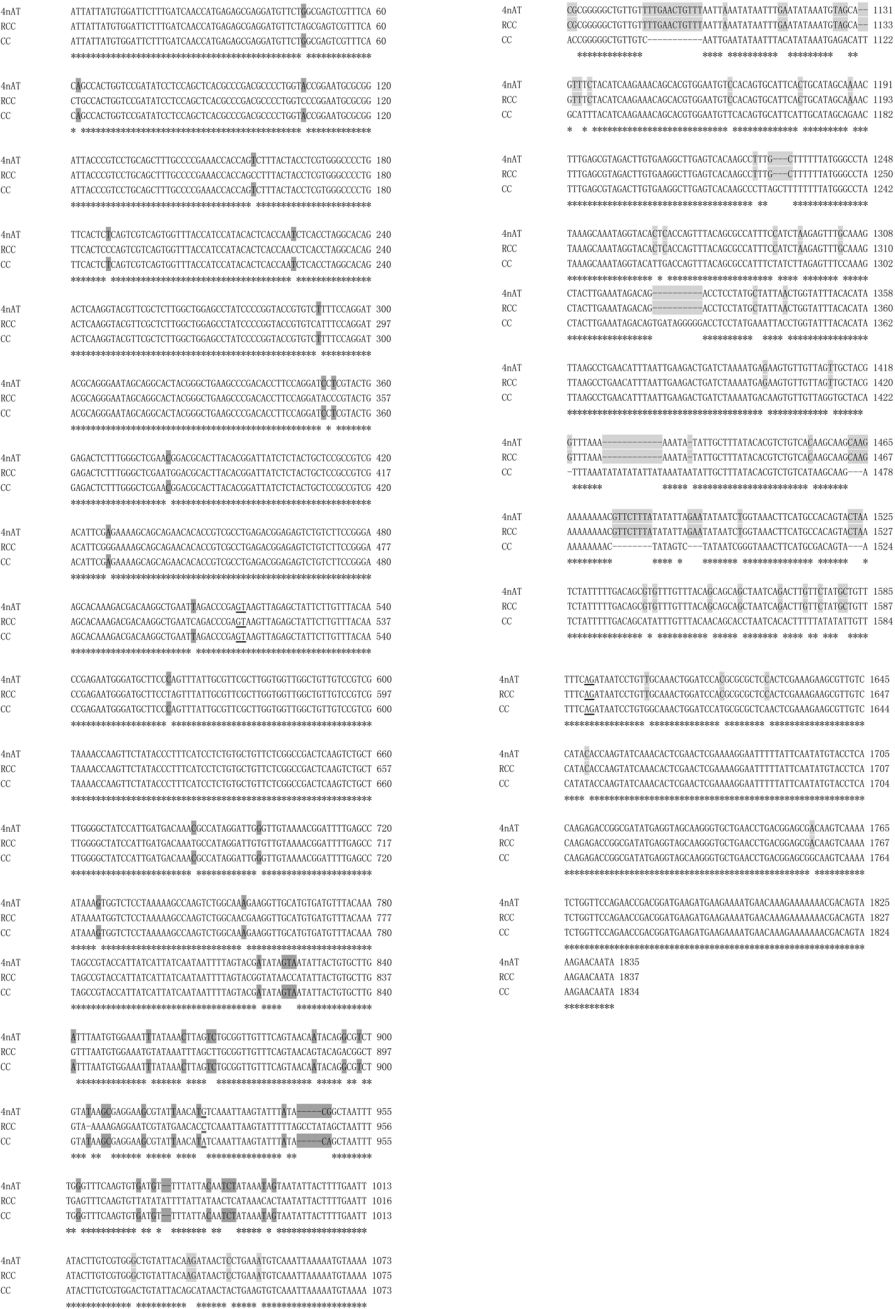
Comparison of recombinant *HoxC9a* gene in 4nAT with *HoxC9a* gene in RCC and CC. Except for one nucleotide marked with the double underscore “=“, which was inconsistent with both RCC and CC, the former part of nucleotide sequence of the recombinant *HoxC9a* gene that occurred in 4nAT was consistent with CC (position: 1–1026); the latter half was consistent with RCC (position: 1027–1825). The boundary bases of introns were underlined with “GT-AG”; “*” meant that the nucleotide was identical at this site

DNA fragments (~ 1800 bp) were amplified from RCC, CC, and 4nAT using the primer pair *HC9aF-HC9aR*. The full-length *HoxC9a* gene in RCC, CC, and 4nAT was 1837 bp (GenBank no: MN584925), 1834 bp (GenBank no: MN584926) and 1835 bp (GenBank no: MN584927), respectively. The homeodomain sequence of the *HoxC9a* gene was identical to the homeobox fragment *HoxC9a* isolated in the PCR survey. Interestingly, in 4nAT, a new fragment of the *HoxC9a* gene was found, whose former part of nucleotide sequence was identical to that of the CC and the latter part was identical to that of RCC; the position of the exchange recombination occurred in the intron region (Fig. 3).

### Hybrid identification

**Fig. 4:**
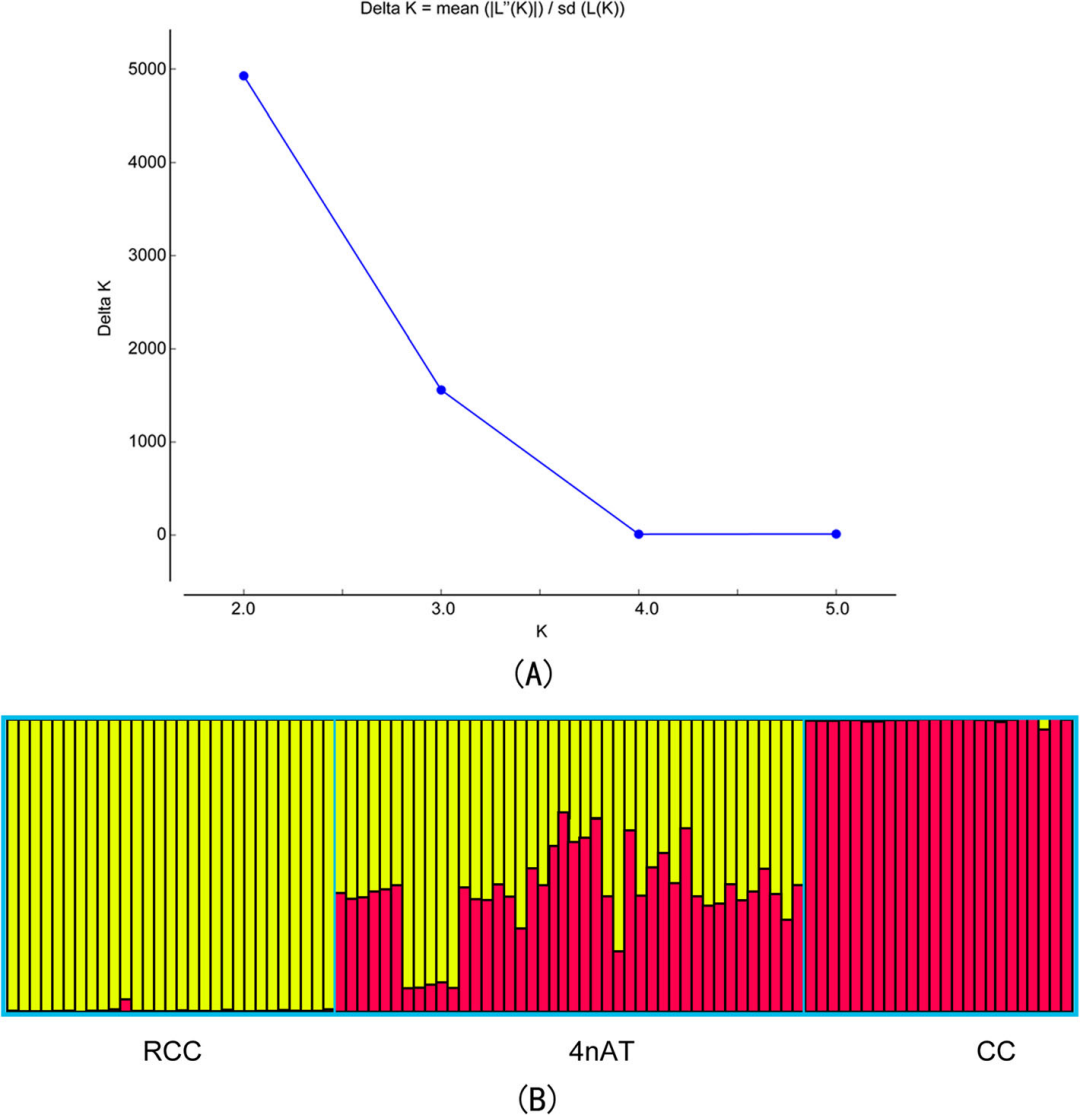
Genetic composition of RCC, CC and 4nAT. STRUCTURE displayed for the optimal number of clusters (K = 2) (**a**). RCC cluster was yellow and CC cluster was Red (**b**)

To determine if 4nAT was hybridized from RCC and CC, we examined the genomic composition of RCC, CC, and 4nAT using STRUCTURE. The genetic composition estimation was consistent with our sample collection. The two parental lines (RCC and CC) corresponded to the two clusters, and they were assigned exclusively to one of the two clusters (Fig. 4). All 4nAT individuals showed mixed ancestries (Fig. 4). This suggested that the 4nAT individuals genetically originated from RCC and CC.

### Phylogenetic relationships

**Fig. 5:**
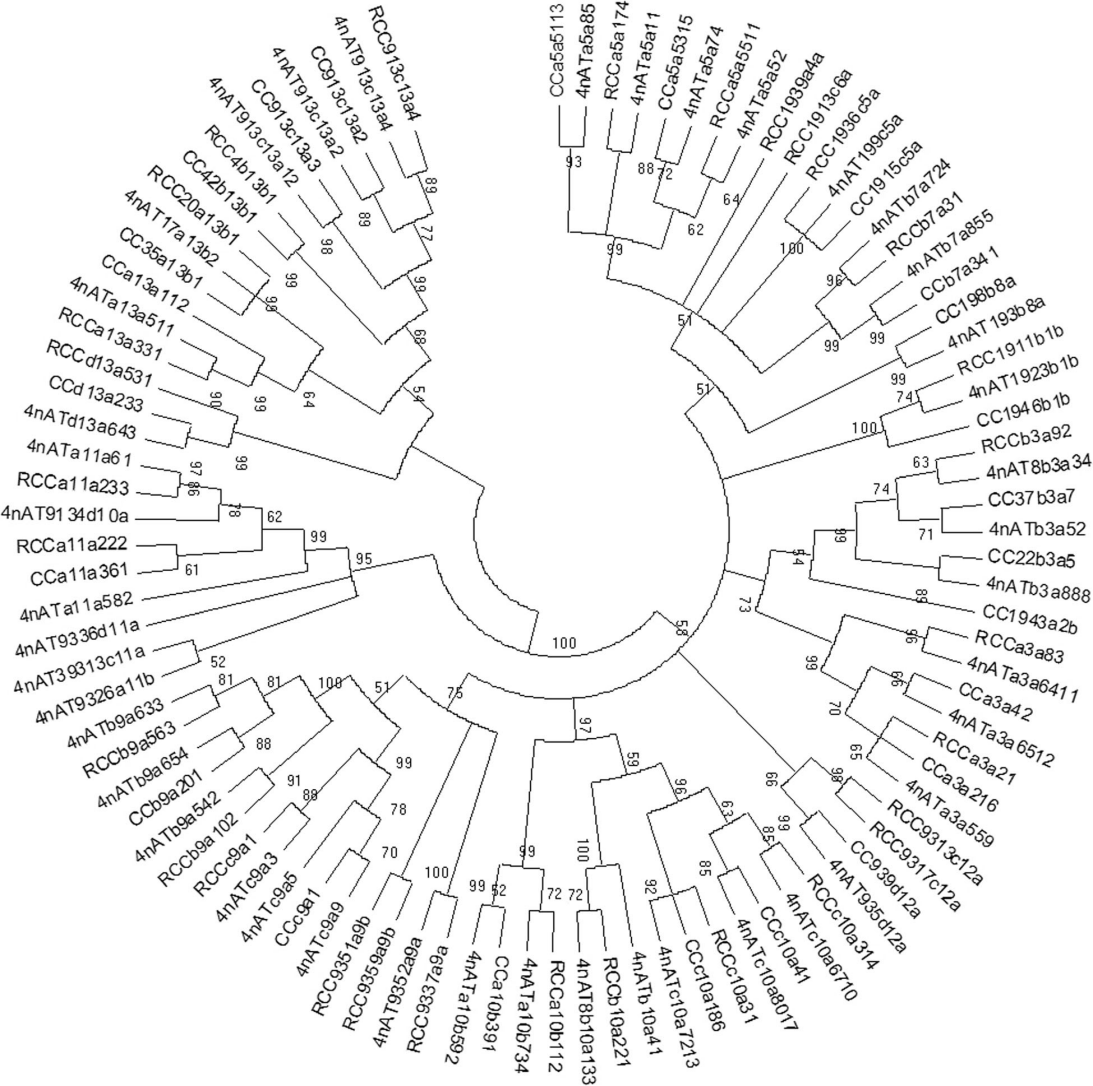
Amino acid-based Maximum Likelihood tree represented the phylogenetic relationships of the putative *Hox* genes obtained from 4nAT, RCC and CC. The number at each node represented the percentage bootstrap value of 1000 replicates. The number on the horizontal line represented the self-expanding value

The identified *Hox* genes belonged to the paralogous groups 1, 2, 3, 4, 5, 6, 7, 8, 9, 10, 11, 12 and 13; however, we did not detect paralogous groups 4 and 6 in CC, paralogous groups 2 and 8 in RCC, and paralogous groups 2, 4 and 6 in 4nAT. The phylogenetic analysis of the protein-coding nucleotide sequences assigned the obtained fragments to the expected orthologous genes among the three species (Fig. 4). Forty-two 4nAT, 29 RCC and 24 CC different subtypes of *Hox* gene fragments were analyzed using the HKY + G + I model in MEGA 5.0 software to estimate the genetic divergence between the species. The phylogenetic tree of the genes was constructed by the ML method, according to genetic distance, and the confidence level (1000 bootstrap replicates) was tested with 1000 replicate samplings (Fig. 5). In this phylogenetic tree, essentially all of the 4nAT *Hox* genes were first aggregated with their related parents, RCC or CC, and then combined with other subtypes of this gene in 4nAT(Fig. 5). The phylogenetic tree showed that the genetic material of 4nAT originated from RCC and CC.

### Analysis of pseudogenes in 4nAT and the related parents

**Fig. 6:**
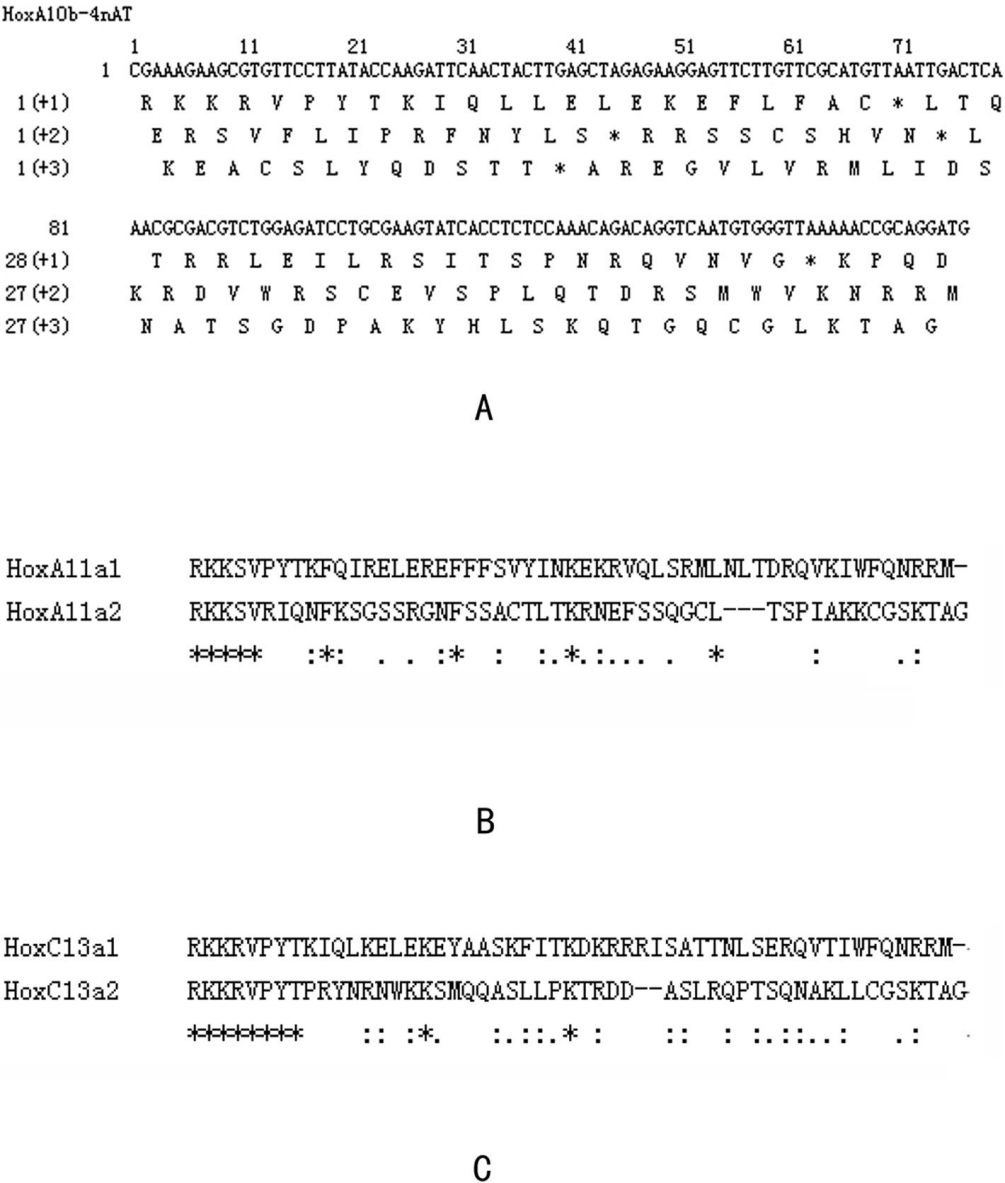
The pseudogenes *HoxA10b* and *HoxC13a2* in 4nAT and the pseudogene *HoxA11a2* in RCC. **a**: listed all the possible amino acid sequences of *HoxA10b* gene in 4nAT which had base deletions. “*” meant that this site was a terminator when a nucleotide was translated into an amino acid. **b** and **c** reflected transcoding mutations in *HoxA11a* and *HoxC13a* genes. The amino acid sequences of *HoxA11a* and *HoxC13a* were expressed as *HoxA11a1* and *HoxC13a1*, respectively. *HoxA11a2* meant that the amino acid sequence of *HoxA11a* gene with the deletion of base, and *HoxC13a2* was on behalf of the amino acid sequence of *HoxC13a* gene with the insertion of base. “*” represented the same amino acid at this site; “:” meant that this site was a conservative mutation; “.” indicated that the position was a semi-conservative mutation; The blank space represented a large variation in the position

A pseudogene was a sequence that existed in the genome of a specific population, and was typically characterized by being very similar to one or more paralogous genes, yet was nonfunctional. This loss of function was due to a failure in transcription or translation, or production of a protein that did not have the same function as the protein encoded by a normal paralog gene. Among the 152 *Hox* gene sequences in 4nAT, we found that the *HoxA10b* and *HoxC13a2* were pseudogenes. Two bases were missing in *HoxA10b* and the length of the *HoxC13a* gene subtype (*HoxC13a2*) with two inserted bases was 161 bp. Among the 88 *Hox* gene sequences of RCC, the *HoxA11a2* was pseudogene. A base-deletion was found in the subtype (*HoxA11a2*) of *HoxA11a* gene and the length of the *HoxA11a2* with base deletion was 158 bp. Using Jellyfish software (Version: V1.4_4383) to translate all the conserved sequence of these *HoxA10b* genes into amino acids, the translation termination appeared in the coded region, in every case. The GC percentage (45.22%) of the pseudogene *HoxA10b* was significantly lower than AT percentage (54.78%). Compared with the amino acid sequence of the *HoxC13a* gene (*HoxC13a1*) and *HoxA11a gene* (*HoxA11a1*), the amino acid sequence of *HoxC13a2* with base insertion and *HoxA11a2* with base deletion had changed greatly after these frameshift mutations, respectively (Fig. 6). The amino acid length of the pseudogenes (*HoxA11a2*: 51; *HoxC13a2*: 52) tended to be lower than that of their counterpart genes (*HoxA11a1*: 53; *HoxC13a1*:53).

### Molecular organization of the *HoxC9a* gene sequence

**Fig. 7:**
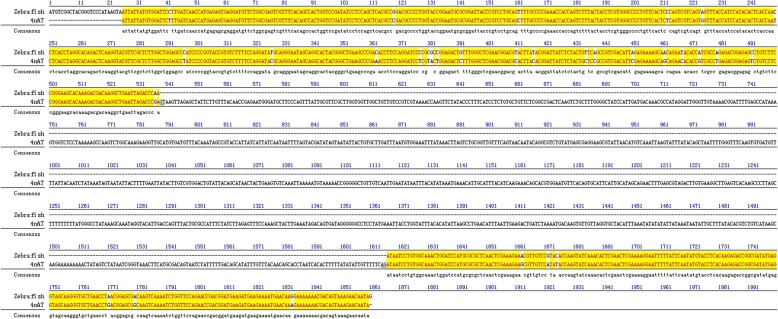
Comparison of *HoxC9a* gene from 4nAT and *HoxC9a* cDNA sequences of zebrafish. The yellow region represented the same sequence in zebrafish and 4nAT

We used the sequence from 4nAT to compare the obtained DNA sequences with the *HoxC9a* cDNA sequence of zebrafish (*Danio rerio*, BC165307) in GenBank (Fig. 7). Both ends of the sequence from 4nAT, RCC and CC resembled the *HoxC9a* cDNA sequence of zebrafish (NM_131528.3), while the intermediate sequence in 4nAT was an extra sequence, compared to the cDNA sequence in zebrafish (Fig. 7). The ends of the extra sequence were ‘GT’ and ‘AG’ using Jellyfish software alignment (539th position and 540th position: GT; 1616th position and 1617th position: AG). The intron junctions were consistent with the “GT-AG” rule in most species [23, 24]. The similarity of the *HoxC9a* gene exon sequence between 4nAT and zebrafish, CC and zebrafish and RCC and zebrafish was 92.70, 92.80 and 91.90%, respectively. The rule also indicated that the intermediate sequence in 4nAT, CC and RCC were an intron, and the ends of the sequence were exon1 and exon2. We obtained the cDNA of the *HoxC9a* gene by excising the intron and ligating exon1 and exon2. Then, the sequence was translated into putative amino acid sequences using Bioedit software (Version 7.0). The putative amino acid sequences of *HoxC9a* in 4nAT, RCC and CC shared approximately 94.6, 93.8, and 94.6% similarity with that of zebrafish, respectively. All of our findings determined the identity of the PCR clones and the exon1-intron-exon2 structure of the *HoxC9a* gene. The full-length *HoxC9a* genes in 4nAT, RCC and CC were obtained, and these sequences were compared and analyzed (Fig. 3). Sequence analysis indicated that the *HoxC9a* gene included exon1, intron and exon2. The intron junctions were consistent with the “GT-AG” rule. The two isoforms of the *HoxC9a* gene in 4nAT were from its original parents. Moreover, a recombinant isoform of the *HoxC9a* gene was found in 4nAT (Fig. 3).

## Discussion

In this study, 29 *Hox* genes were screened in 4nAT and its parents (RCC and CC) using PCR. Among these, 23, 22 and 19 *Hox* genes were detected in 4nAT, RCC and CC, respectively. Compared with the *Hox* genes in zebrafish [25], all of the *Hox* genes detected here had homologous genes in zebrafish. The *Hox* genes in 4nAT, CC and RCC were also found in goldfish with twin tails [26]. In tetraodon nigroviridis [27], fugu rubripes [28], and medaka [29], the identified *Hox* genes did not have complete identity with the *Hox* genes in RCC, CC, and 4nAT. The *HoxB10a* and *HoxD13a* genes were absent from the tetraodon nigroviridis, fugu rubripes, and medaka, but these genes were present in 4nAT and its related parents (RCC and CC). The retention and loss of genes may be associated with the evolutionary process [30]. These data allowed us to study the evolution of 4nAT after the doubling of the *Hox* gene cluster.

Many cloned fragments were obtained from *Hox* genes, such as *HoxA3a*, *HoxA5a* and *HoxC10a*, by PCR amplification. The length of these genes was 149 bp in 4nAT and its parents. In 4nAT, there were four subtypes of *HoxA5a*. Two had the same subtype as the *HoxA5a* gene of RCC, and two had the same subtype as the *HoxA5a* gene in CC. Three subtypes of the *HoxA3a* and *HoxC10a* genes were detected in 4nAT, which also originated from RCC and CC. *HoxA3a*, *HoxA4a* and *HoxB4a* from zebrafish were found in *Oncorhynchus mykiss*, and these genes in rainbow trout had two specific homologous zebrafish genes [31]. Two specific homologous zebrafish genes of the *Hox* gene in RCC and CC were found, and there were more subtypes in 4nAT. These results suggested that genome duplication had occurred in other cyprinid fish. A comparison of the homologous parts of all subtypes of the *Hox* genes in the 4nAT, RCC and CC, showed that the homologous frame parts of the *Hox* genes in the 4nAT did not exhibit large-scale base mutations after doubling. The sequence of this segment was stable. The *Hox* genes in the 4nAT were more complex and abundant than those in RCC and CC.

After genome doubling, the new genome could face selection pressure, which could result in the evolution of new features. Genome duplication produced abundant genomic DNA, so the hybrid maintained the dosage balance or rapidly stabilized the duplicated genomes via retention/exclusion of redundancy. Lynch et al. [32] suggested three possible outcomes from the evolution of duplicate genes: non-functionalization, neo-functionalization and subfunctionalization. Duplicated genes were either retained without changes, mutated into other genes with new functions or degenerated into nonfunctional pseudogenes. We found that the *HoxA10b* gene in 4nAT exhibited two base deletions, while the *HoxC13a* gene had two base insertions. In the *Hox* gene sequence of RCC, a base deletion was also found in the *HoxA11a* gene sequence. However, whether the *Hox* gene in CC also had this base variation remained unknown. The occurrence of these pseudogenes (*HoxA10b*, *HoxA11a* and *HoxC13a*) helped to reduce the pressure created by genome doubling. This pressure was consistent with the expectation that there were *Hox* clusters in the 4nAT genome that had lost functional *Hox* genes due to the reduction of redundancy of the *Hox* genes due to the polyploidization event. In the hybridization process, 4nAT required genetic recombination, mutation, and pseudogenization to reduce the amount of incompatible genetic material and improve fertility [13]. Thus, we established an allotetraploid fish lineages [7]. The results indicated that the genome after doubling could reduce redundant genes by formation of pseudogenes, as indicated by the *Hox* gene in 4nAT. Characterization of the *Hox* gene clusters in allotetraploid hybrids increased understanding of the evolutionary process which occurred after *Hox* gene doubling.

We cloned the full-length sequence of the *HoxC9a* gene, and its structure was divided into three parts: exon1, intron and exon2. This result was consistent with the *Hox* structure described in previous reports [33, 34], in which the bases of the introns conformed to the GT-AG rule. Analysis of the *HoxC9a* genes in RCC, CC and 4nAT, showed that genetic recombination occurred in 4nAT, and the intron region occurred in the recombination region. The former part was consistent with CC, and the latter part was consistent with RCC. This recombinant gene indicated that 4nAT underwent meiosis, alien chromosome synapsis and exchange recombination. The exchange recombination provided a genetic basis for the variation and diversity of the 4nAT and provided raw materials which might facilitate species adaptation and evolution.

In summary, we investigated the gene organization and structures of *Hox* in 4nAT and its parents. There was significant variation in *Hox* genes, which enabled rapid genomic evolution in 4nAT after hybridization and polyploidization. In fish, hybridization and polyploidization could help drive speciation by changing genome structures and generating new genes.

## Conclusions

We found four copies of *Hox* genes in the 4nAT, two copies in RCC and two copies in CC. Obvious variation and pseudogenization were found in some *Hox* genes of the 4nAT. These results revealed the effects of polyploidization on the organization and evolution of *Hox* gene clusters in fish, and helped to clarify aspects of vertebrate genome evolution.

## Methods

### Samples and ethics statement

RCC, CC and 4nAT were obtained from State Key Laboratory of Developmental Biology of Freshwater Fish, Hunan Normal University, Changsha, Hunan, China. The procedures were conducted in accordance with approved guidelines. Fish were housed in open pools (0.067 ha) with a suitable pH (7.0–8.5), water temperature (22–24 °C), and dissolved oxygen content (5.0–8.0 mg/L) and with adequate food. The fish used for blood samples were anesthetized with 100 mg/L MS-222 (Sigma-Aldrich, St. Louis, MO, United States).

### DNA extraction

One-year-old 4nAT, one-year-old RCC, and one-year-old CC, were randomly selected. Peripheral blood was collected from the caudal vein of each fish (for total genomic DNA extraction). Total genomic DNA was isolated from blood cells following the manufacturer’s instructions (Sangon, Shanghai, China). The concentration and quality of DNA was assessed using agarose gel electrophoresis.

### PCR amplification, cloning, and sequencing

PCR amplification of 108 bp of the highly conserved homeobox PG1–9 was performed using a degenerate homeobox primer pair: forward primer [5′-GAA TTC CAC TTC AAC (C/A)(G/A)(C/G) TAC CT-3′] and reverse primer [5′-CAT CCT GCG GTT TTG GAA CCA NAT-3′]. PCR amplification of 159 bp of the highly conserved homeobox PG9–13 was performed using a degenerate homeobox primer pair: forward primer [5′-CGA AAG (C/A)G(N/C) GT(N/C) CC(N/C) TA(T/C) AC-3′] and reverse primer [5′-CAT CCT GCG GTT TTG GAA CCA NAT-3′] as described by Amores et al. (2004) [35]. We used Primer Premier 5.0 to design a pair of degenerate primers [HC9aF, 5′-ATT ATT ATG TGG A(C/T) T C(C/T) T TGAT-3′; HC9aR, 5′-TA(C/T) TGT TC(C/T) TT(A/G) CTG TCG TT(C/T) T-3′] based on nucleotide and amino acid sequences of *HoxC9a* in zebrafish (*Danio rerio*), pufferfish species (*Takifugu rubripes* and *Tetraodon nigroviridis*) and medaka (*Oryzias latipes*). Amplification was carried out in a 20 μl reaction volume containing 2.0 μl 10 × PCR buffer, 1.6 μl 25 mM MgCl_2_, 1.6 μl 2.5 mM dNTPs, 0.6 μl 10 μM forward and reverse primers, 0.4 μl 2.5 U/μl Taq DNA polymerase (Tiangen, Beijing, China), and approximately 100 ng genomic DNA. PCR was carried out under the following standard cycle: one denaturation step at 94 °C for 5 min, 30 cycles of 94 °C for 30 s, 55 °C for 30 s and 72 °C for 1 min, followed by a final extension step at 72 °C for 5 min. All amplified fragments were purified by a gel extraction kit and ligated into a *pMD*18-T vector (Takara, Dalian, China) following the manufacturer’s protocol. The plasmids were transformed into *E. coli* DH5α. The clones were identified by PCR amplification and sequenced by an automated DNA sequencer (ABI PRISM 3730).

### Analyses of PCR fragments

All raw sequence data were verified manually from electropherograms using the Chromas version 1.62 programs (Technelysium, Australia). The 108 bp and 149 bp sequences of the PG1–9 and PG9–13 homeoboxes, respectively, were screened for *Hox* genes using BLAST searches against NCBI. The ~ 1500 bp long sequences of *HoxC9a* were screened for *Hox* genes using BLASTX and BLASTN to determine their identity. The alignment of each PCR fragment to one of the paralog groups (*Hox1*–*Hox13*) was initially determined based on nucleotide and amino acid sequence similarities to published *Hox* sequences using BLAST (http://www.ncbi.nlm.nih.gov/BLAST/).

### Sequence comparison and analysis

Sequence homology and variation among the fragments amplified from RCC, CC and 4nAT were analyzed in Bioedit (Version 7.0). Partial DNA sequences for each gene were verified via a BLASTX search. To increase the probability of detecting duplicated paralogs and avoiding PCR errors, we sequenced 30 clones of each gene for RCC, CC and 4nAT. BLASTX and BLASTN searches of the GenBank database were initially made to determine the identity of the PCR clones. Then, the DNA sequences were compared with the *HoxC9a* cDNA sequence of zebrafish (*Danio rerio*, BC165307) in GenBank using the Jellyfish software to identify the exon1-intron-exon2 structure of the *HoxC9a* gene. The intron junctions were consistent with the “GT-AG” rule in most species and the rule was used to reconfirm the position of the intron of the *HoxC9a* gene. We obtained the cDNA of the *HoxC9a* gene by excising the intron and ligating exon1 and exon2, and then the sequence was translated into putative amino acid sequences using Bioedit software (Version 7.0). The work above was performed to determine the identity of the PCR clones and the exon1-intron-exon2 structure of the *HoxC9a* gene.

### Genetic composition analysis

To determine if 4nAT individuals genetically originated from RCC and CC, we inferred genomic composition with *Hox* gene haplotypes using STRUCTURE [36]. We ran STRUCTURE for K-values varying from 1 to 6, with 20 replicates at each K. Each replicate run had a total of 100,000 iterations, with the first 50,000 discarded as burn-in. The optimal K-value was 2 (Fig. 4a) in the analysis with STRUCTURE HARVESTER [37]. We combined the 20 runs at K = 2 using CLUMPP [38] and conducted a graphical display by DISTRUCT [39].

### Phylogenetic analysis

The *Hox* gene sequences were aligned using ClustalX software [40] and a Maximum Likelihood (ML) phylogenetic tree was constructed using MEGA 5.0 software [41]. We assigned PCR fragments based on the identity of the subtree in which they were located. Phylogenetic analysis was performed by ML methods and the best-fitting nucleotide substation model with the lowest BIC score was determined by using MEGA5 [42]. Conserved regions were determined using the Gblocks program. ML analyses were performed using the GTR + G model, and the robustness of the tree topology was assessed with 1000 bootstrap replicates [43].

## Data Availability

The datasets supporting the conclusions of this article were available in the GenBank repository with access No. MN584925-MN584927.

## Abbreviations

2nRCF_1_: The first-generation diploid hybrids of RCC (♀) × CC (♂)
2nRCF_2_: The self-mating of 2nRCF_1_
4nAT: Allotetraploid hybrids
CC: Common carp
RCC: Red crucian carp
